# The Development of a Web-Based Application to Support Informal Caregivers of Individuals With Head and Neck Cancer: Human-Centered Design Approach

**DOI:** 10.2196/81896

**Published:** 2026-04-08

**Authors:** Awais Ahmad, Åsa Cajander, Ulrica Langegård, Birgitta Johansson, Anna Henriksson, Mona Pettersson, Waqas Ahmad, Ylva Tiblom Ehrsson

**Affiliations:** 1 Department of Information Technology, Uppsala University, Lägerhyddsvägen 1, Uppsala, 75237, Sweden, 46 734697404; 2 Department of Oncology, Sahlgrenska University Hospital, Gothenburg, Sweden; 3 Institute of Health and Care Sciences, University of Gothenburg, Gothenburg, Västra Götaland, Sweden; 4 Department of Immunology, Genetics, and Pathology, Uppsala University, Uppsala, Sweden; 5 Department of Women’s and Children’s Health, Uppsala University, Uppsala, Sweden; 6 Department of Public Health and Caring Sciences, Uppsala University, Uppsala, Sweden; 7 Department of Software Engineering, A2Z Technology, Stockholm, Sweden; 8 Department of Surgical Sciences, Uppsala University, Uppsala, Sweden

**Keywords:** human-centered design, designing for well-being, eudaimonic motivation, head and neck cancer, informal caregivers

## Abstract

**Background:**

Informal caregivers (ICs), often family members or close friends, provide essential support to individuals with head and neck cancer. However, they are frequently unprepared for the emotional, practical, and medical challenges involved. Web-based applications offer promising opportunities to support ICs, but their long-term adoption and acceptance remain uncertain.

**Objective:**

This paper presents the development of Carer eSupport, a web-based application to support ICs’ well-being and preparedness for caregiving. We detail the design and functionality of the Carer eSupport application and explain how it responds to both the functional and psychological needs of ICs. Additionally, we report findings from the pilot study and highlight the initial challenges ICs faced when engaging with the application, along with the strategies used to overcome them.

**Methods:**

The study involved a multicenter research trial across ear, nose, and throat clinics and oncology and radiotherapy clinics at 4 university hospitals in Sweden. The application was developed through 3 human-centered design (HCD) iterations involving ICs, health care professionals, and researchers in human-computer interaction and cancer care.

**Results:**

The results present an overview of the current version of Carer eSupport (developed during the third design iteration), with a focus on features that address the psychological needs of ICs, including competence, autonomy, and a sense of connection to others. The pilot study achieved a 66.7% (20/30) consent rate, a 75% (9/12) successful login rate among participants, and a 13.3% (4/30) attrition rate, meeting the established criteria. The pilot study confirmed the application’s readiness for further evaluation in an ongoing randomized controlled trial. It also identified challenges, including the time constraints of ICs, login and authentication issues, limited IT infrastructure, and gaps in digital literacy.

**Conclusions:**

Findings from the HCD process and pilot study indicate that a personalized, interactive application like Carer eSupport can provide meaningful support for ICs of individuals with head and neck cancer. The integration of HCD and health care science offers early guidance for developing digital tools that are both evidence-based and empathetic, with potential relevance beyond caregiving contexts.

## Introduction

### Background

Health care systems worldwide face mounting pressure as they contend with limited resources and the increasing care needs of an aging population [1,2]. These strains are particularly evident in home-based care, where access to professional support is often limited. In cancer care, the shift from hospital-based treatment to outpatient and home settings has transferred much of the day-to-day responsibility to family members and close friends [3,4]. These individuals, commonly referred to as informal caregivers (ICs), provide unpaid support and are often the primary source of informal care for people with cancer [5,6]. They often struggle to navigate their roles because they lack access to the essential information, guidance, and resources needed to provide adequate care at home [7-10].

The emotional and physical toll of caregiving can be significant, affecting ICs’ overall well-being. It often leads to mental health challenges such as anxiety, depression, and symptoms of post-traumatic stress [11-15]. Over time, ICs may begin to lose a sense of their own identity as their lives become increasingly centered around the illness of their patients. These difficulties are made worse when the support systems and information needed to manage care at home are limited or hard to access [16]. Therefore, ICs often express a strong need for external support and guidance to feel more confident and capable in their roles [17,18]. This is where the concept of preparedness for caregiving becomes relevant. Preparedness refers to ICs’ perceived ability to provide physical and emotional support while also managing the stress of caregiving [19]. Higher levels of preparedness have been linked to lower caregiver burden and better outcomes for both ICs and patients [3]. Given these challenges, digital tools such as web-based applications offer promising opportunities to support ICs. These eHealth systems can serve as accessible platforms for delivering tailored information, emotional support, and practical guidance to those providing cancer care at home [12,16]. However, acceptance and use of eHealth systems remain uncertain, highlighting the need for further research on design strategies that ensure the meaningful involvement of relevant stakeholders throughout the development process [20-23].

Human-centered design (HCD) is a foundational approach for developing technology in the human-computer interaction (HCI) field. HCD emphasizes placing users’ needs, preferences, and contextual factors at the core of the design and development process to create effective and user-empowering systems [23]. Even though HCD is widely accepted and used, it often falls short of meeting users’ specific needs, making them feel disconnected and unsupported [24,25]. One major reason for this gap is how HCD is applied. When used in a limited or inconsistent way, especially without genuine and ongoing stakeholder involvement, it can become more of a label than a clear design method. Consequently, although HCD holds significant potential to enhance the usefulness and user-friendliness of technology, its impact remains limited when it is not implemented consistently and systematically in practice [25-28].

The HCD process has primarily focused on usability and functionality, but there is growing recognition that technology should also support users’ well-being [29-34]. Well-being is a multifaceted concept that includes physical health, mental and emotional stability, social relationships, economic security, access to education and health care, environmental conditions, cultural aspects, personal safety, and overall life satisfaction [35]. People tend to use technologies that satisfy their functional needs and enhance their physical and psychological well-being [34]. In the field of psychology, well-being is often understood in two ways. Hedonic well-being is about feeling good, seeking pleasure, and avoiding discomfort. On the other hand, eudaimonic well-being is about personal growth, having a sense of purpose, and reaching one’s full potential [36,37]. Eudaimonic motivation is a person’s inner drive to grow, learn, and seek meaningful experiences in life [36]. Research on hedonic aspects is more established. For instance, the well-known extended Unified Theory of Acceptance and Use of Technology (UTAUT2) model evaluates technology acceptance by integrating hedonic motivation as a key factor in technology acceptance and use [38]. However, there is a noticeable scarcity of research that explicitly examines how eudaimonic motivation can be systematically integrated into the HCD process [39-42].

Some recent studies have sought to evaluate eudaimonic motivation quantitatively [33,42,43]. For instance, Woźniak et al [42] combined insights from psychology to develop the Eudaimonic Technology Experience Scale, which aims to quantify the meaningful interaction of users. Similarly, Kuriakose and Nagasubramaniyan [39] extended UTAUT2 to assess the acceptance of digital entertainment applications. Despite some attempts to evaluate eudaimonic motivation quantitatively, a significant gap remains in the qualitative understanding of how eudaimonic motivation influences technology acceptance and use, particularly in complex and emotionally sensitive domains such as caregiving for relatives with serious diseases. Moreover, these studies focus exclusively on the evaluation phase of the HCD process. This study integrates eudaimonic perspectives from the early stage of the HCD process, with the aim of supporting users’ psychological well-being.

### The Carer eSupport Project

Carer eSupport is a multidisciplinary project combining HCI, software engineering, cancer nursing, and medical and health care science expertise. It seeks to develop a web-based application that improves ICs’ preparedness for caregiving and promotes their overall well-being [3,44]. By equipping ICs with targeted, accessible, and user-friendly resources, the project aims to reduce caregiver burden and positively support the overall health of individuals with head and neck cancer (HNC).

We applied the integrated HCD process with a eudaimonic perspective to guide the development and evaluation of Carer eSupport. The HCD process involved 3 iterative cycles, engaging ICs, health care professionals (HCPs), an expert group of ICs, and technology designers and developers. The collaboration of different stakeholders enabled us to continuously refine the application to understand and address the practical and psychological needs of ICs. The project is currently conducting a randomized controlled trial (RCT) to evaluate the effects of Carer eSupport on ICs’ self-reported preparedness for caregiving, caregiver burden, and well-being, compared to support as usual [44]. A pilot study was conducted at the early stage of the RCT to investigate the feasibility of the procedures and the current version of Carer eSupport. This paper presents the results of the pilot study, which determined whether it was appropriate to proceed with the full-scale RCT.

### Study Aim

This paper aims to present how a web-based application can be designed to support ICs’ well-being and preparedness for caregiving through the HCD process. We detail the design and functionalities of the Carer eSupport application and explain how it responds to both the functional and psychological needs of ICs. By integrating HCD with a eudaimonic perspective, this research also intends to provide insights into design approaches that prioritize user well-being in web-based application development.

Additionally, we report findings from the pilot study and highlight the initial challenges ICs faced when engaging with the application and the strategies used to overcome these issues.

## Methods

### Development of Carer eSupport

#### Human-Centered Design Process

We conducted 3 iterations of the HCD process to develop the Carer eSupport application. HCD is an iterative approach that prioritizes users and other stakeholders at the core of the design process [45]. This iterative process ensured that the application addressed the needs of ICs by incorporating their direct feedback in different design phases. As highlighted in previous research [46,47], continuous refinement based on user input enhanced usability, relevance, and support for both the practical and psychological challenges of caregiving.

The first step in the HCD process involves understanding users and their context through comprehensive research methods, including interviews, observations, surveys, and focus groups, to define user requirements. Thereafter, the design team defines the problem and establishes specific requirements based on these insights. They then produce design solutions through brainstorming and concept development, which are evaluated through user testing to assess usability, functionality, and overall satisfaction. The design is iteratively refined based on feedback until the final product effectively meets user needs and expectations [48].

As a multidisciplinary project combining health care research and computer science expertise, we integrated health care research methods, such as feasibility studies and RCTs, with HCD to develop and evaluate Carer eSupport. In the following sections, we detail the 3 iterations that shaped the final version of the application. Findings from the first and second iterations have been presented in detail elsewhere [15,49]. This study primarily focuses on the third iteration, with its key findings presented in the Results section. Table 1 provides an overview of all 3 HCD iterations, including the major activities conducted and a summary of their outputs during the design and development of Carer eSupport.

**Table 1.** Overview of the human-centered design (HCD) iterations in the development of Carer eSupport.

HCD iterations	Key activities	Outputs
1: Prototype design	Focus groups Prototyping Developed the initial content of Carer eSupport Evaluation with the expert group of ICs^a^	Interactive prototype and initial content of Carer eSupport
2: Feasibility study	Developed the initial version of Carer eSupport Mixed methods evaluation (surveys, application usage logs, interviews) Extended the UTAUT^b^ model with eudaimonic motivation	Confirmation of the feasibility and acceptability of Carer eSupport and suggestions for improvements before the randomized controlled trial
3: Pilot study	Developed the current version of Carer eSupport Pilot study evaluation	Confirmation to continue the full-scale randomized controlled trial and improvements of its procedures

a ICs: informal caregivers.

b UTAUT: Unified Theory of Acceptance and Use of Technology.

#### Iteration 1: Prototype Design

##### Understanding ICs’ Needs and Specifying Their Requirements

To identify the challenges and needs of ICs, we conducted focus groups with ICs and HCPs. These sessions provided essential insights into user preferences and the functional requirements for the Carer eSupport application. In this iteration, we explored both hedonic and eudaimonic aspects of well-being. Using a deductive thematic analysis, we examined factors such as virtue, autonomy, competence, relatedness, achievements, and engagement derived from the focus group data. Our findings indicate that pleasure, as a component of hedonic motivation, is not applicable in the caregivers’ context—a point we discussed in detail in our previous study [49].

Three main stakeholder groups took part in this phase. ICs shared their experiences and highlighted the complexity of caregiving, stressing the need for support in patient care and their well-being. HCPs, including nurses, doctors, dietitians, and speech therapists, ensured the application was clinically relevant. A caregiver expert group also reviewed the content in several sessions and gave feedback that helped refine and improve the application. This expert group comprised ICs who had more extensive and continuous involvement in the design process, working closely with the research team to review, discuss, and refine both the application structure and its content. Their role differed from that of ICs participating in focus groups or later study phases, such as the feasibility study and the RCT, as they were engaged in repeated sessions and contributed more deeply to design decisions. In recognition of their sustained contribution and level of engagement, members of the expert group were financially compensated for their participation.

##### Prototype Development

Building on these insights, we developed an interactive prototype of Carer eSupport using Figma, a collaborative tool for designing and evaluating responsive prototypes [50]. The prototype was created to address the identified needs and preferences of ICs. It included key functions such as information on HNC treatment, resources to support ICs’ well-being, a web-based discussion forum, and an online meeting space. The project team reviewed all the functionalities collaboratively to ensure they aligned with user requirements.

##### Prototype Evaluation

The caregiver expert group preliminarily evaluated the prototype. To collect their feedback, 2 meetings were organized, 1 online and 1 in person. During the online meeting, 2 health care researchers and the HCI researcher presented the initial prototype, along with early versions of the portal content, to the expert group.

The second meeting was a full-day, in-person session held at Uppsala University. During the first 2 sessions, the group was introduced to the application’s video content, including demonstrations, lectures, and presentations. These sessions enabled discussion of the relevance and usefulness of the materials for caregiving. In a subsequent session, the prototype’s main functions were demonstrated, which supported more detailed feedback on usability and navigation.

The expert group reported overall satisfaction with the interface but also identified areas for improvement in both content and functionality. Their suggestions were incorporated into the design, and the prototype was refined before proceeding to the feasibility study in the second iteration of the HCD process.

### Iteration 2: Feasibility Study

#### Development of the Initial Version of Carer eSupport

Based on feedback from the first iteration, the initial functional version of Carer eSupport was developed and structured around 3 major categories. The first category provides educational content on HNC diagnosis, treatment, and management. All the content was provided through text and video to facilitate diverse user needs. The second category provides critical interventions and practical advice on managing symptoms, side effects, and caregiving challenges, focusing on areas such as oral health care, nutrition, and fatigue. The third category, Self-Care for ICs themselves, provides resources to support ICs’ well-being by addressing issues such as everyday life support, sleep difficulties, tiredness, and a diary for finding balance. All content was curated in collaboration with HCPs and researchers specialized in HNC to ensure clinical accuracy and relevance.

#### Evaluation of Initial Version Carer eSupport

A feasibility study using a mixed methods approach was conducted in accordance with the Medical Research Council’s guidelines for developing and evaluating complex interventions [51]. Quantitative data, such as survey responses and application log usage, were combined with qualitative insights from semistructured interviews [44]. The evaluation confirmed the application’s relevance, acceptability, and usability, with recommendations for further refinement. Positive feedback from ICs also highlighted the application’s potential to support their caregiving responsibilities and to improve their well-being. We also extended the UTAUT model in the feasibility study by integrating the eudaimonic motivation construct and proposed an extended UTAUT framework for analyzing the application’s impact on ICs’ well-being.

### Iteration 3: Pilot Study

In the third iteration of the HCD process, building on insights from previous iterations, the current version of Carer eSupport was developed and is currently being evaluated through an ongoing RCT [44]. Widely considered a gold standard for examining the effectiveness of interventions in health care and behavioral research [52], RCTs use a rigorous methodology where study participants are randomly assigned to either an intervention group or a control group. The random allocation of participants ensures an unbiased comparison by minimizing the influence of confounding variables and helps researchers establish a causal relationship between the intervention and its outcomes [53]. Before proceeding to a full-scale RCT, the last step was to conduct a pilot study to evaluate feasibility, refine procedures, and assess the application’s potential for large-scale implementation. Although this paper outlines the entire HCD process for designing and developing Carer eSupport, it primarily focuses on the outcomes of the third iteration, the pilot study.

### Procedures

In this section, we present the procedures for the RCT and pilot study conducted in the third iteration of the HCD process. Patients aged 18 years or older with HNC scheduled to receive radiotherapy with or without surgical or oncology medical treatment were identified and screened by nurses from ear, nose, and throat or oncology and radiotherapy clinics. Eligible patients were approached by a clinical or research nurse, in person or by telephone, and were provided with detailed information about the study. Consent was obtained to contact an IC nominated by the patient and access information from the medical record about the HNC diagnosis, treatment, and outcome.

Once the patient provided informed consent, the identified IC was contacted using their preferred method (in person, by telephone, or by email) and was provided comprehensive information about the study before obtaining their oral and written consent. Enrolled ICs were then provided with credentials to complete an internet-administered baseline assessment. These credentials included a web address, a unique user ID, a temporary password, and a detailed user guide with screenshots to facilitate navigation. After completing the baseline assessment, ICs were randomly assigned to 1 of 2 participant groups: (1) the Carer eSupport plus standard care group or (2) the standard care-only group. ICs in the Carer eSupport plus standard care group received additional login credentials to engage with the application. The data collection methods, including the primary outcome (preparedness for caregiving), secondary outcomes, and assessment points, are presented in detail in the study protocol [44].

### Pilot Study Analysis

A pilot study was conducted to confirm the feasibility of the study procedures and the Carer eSupport application. Pilot studies in RCTs are essential for gauging the likely success of the main trial and, if needed, refining its design [54]. Thirty ICs participated in this phase. Feasibility was analyzed using predefined progression criteria to determine whether the study could proceed to a full-scale RCT. These criteria were as follows: at least 45% of eligible ICs providing written consent, at least 50% actively using the Carer eSupport application, and an attrition rate below 30% one month after randomization. Meeting these benchmarks would allow the study to proceed to a full-scale RCT; otherwise, recruitment would be discontinued. If the criteria were not met, ICs assigned to the Carer eSupport plus standard care group would retain access to the application for the entire 18-week intervention period. However, no further follow-up assessments would be conducted. Please refer to the study protocol for more detailed insights into the study methods and procedures [44].

In addition, qualitative data were collected and analyzed during the pilot study. One week after the login details were distributed, ICs who had not logged in were contacted by telephone to check whether they required assistance. Two researchers conducted these calls and took detailed notes, which were discussed jointly to ensure a shared understanding of the content. One researcher then analyzed the notes using thematic analysis following Braun and Clarke’s framework [55]. The analysis involved familiarization with the data through repeated readings, generation of initial codes, and organization of these codes into broader themes. Themes were iteratively reviewed and refined to ensure they were grounded in the data and aligned with the study aims. The final set of themes was discussed and agreed upon within the research team, which strengthened the credibility and trustworthiness of the analysis.

### Ethical Considerations

All research procedures in the project were approved by the Swedish Ethical Review Council (Dnr: 2020-04650), and the feasibility evaluation is also registered on ClinicalTrials.gov (NCT05028452). The feasibility study was registered at ClinicalTrials.gov (NCT05028452), and the RCT is also registered at ClinicalTrials.gov (NCT06307418).

Informed consent was also obtained from patients, their ICs, and HCPs before their enrollment in the study. Before participating, ICs received a written information letter outlining the study’s purpose and data collection procedures and signed an informed consent form. In addition, a verbal explanation was provided prior to the focus groups and interviews to ensure participants had a clear understanding of the study. No compensation was provided to the participants in this study.

Safeguarding anonymity and privacy was a key ethical priority. Participants were informed that no identifiable personal information would be disclosed in any publications or reports. All interviews were audio- and video-recorded and transcribed verbatim. The recordings were stored securely in Uppsala University’s database and were accessible only to authorized members of the research team.

## Results

### Overview

This section first gives an overview of the current version of the Carer eSupport application, developed during the third iteration of the HCD process. We present the application’s main modules and explain how the eudaimonic perspective is integrated into them. It serves as a practical example of how a web-based application is developed using an iterative HCD process, with a special focus on eudaimonic motivation to enhance ICs’ well-being and improve their preparedness for caregiving. We then present the findings from the pilot study, which indicate that the predefined progression criteria were achieved, supporting the continuation to a full-scale RCT. Finally, we present the initial challenges ICs faced during the pilot study phase when using the application and describe the strategies implemented to improve its usability and accessibility.

### Carer eSupport Application

#### Overview

**Figure 1.**
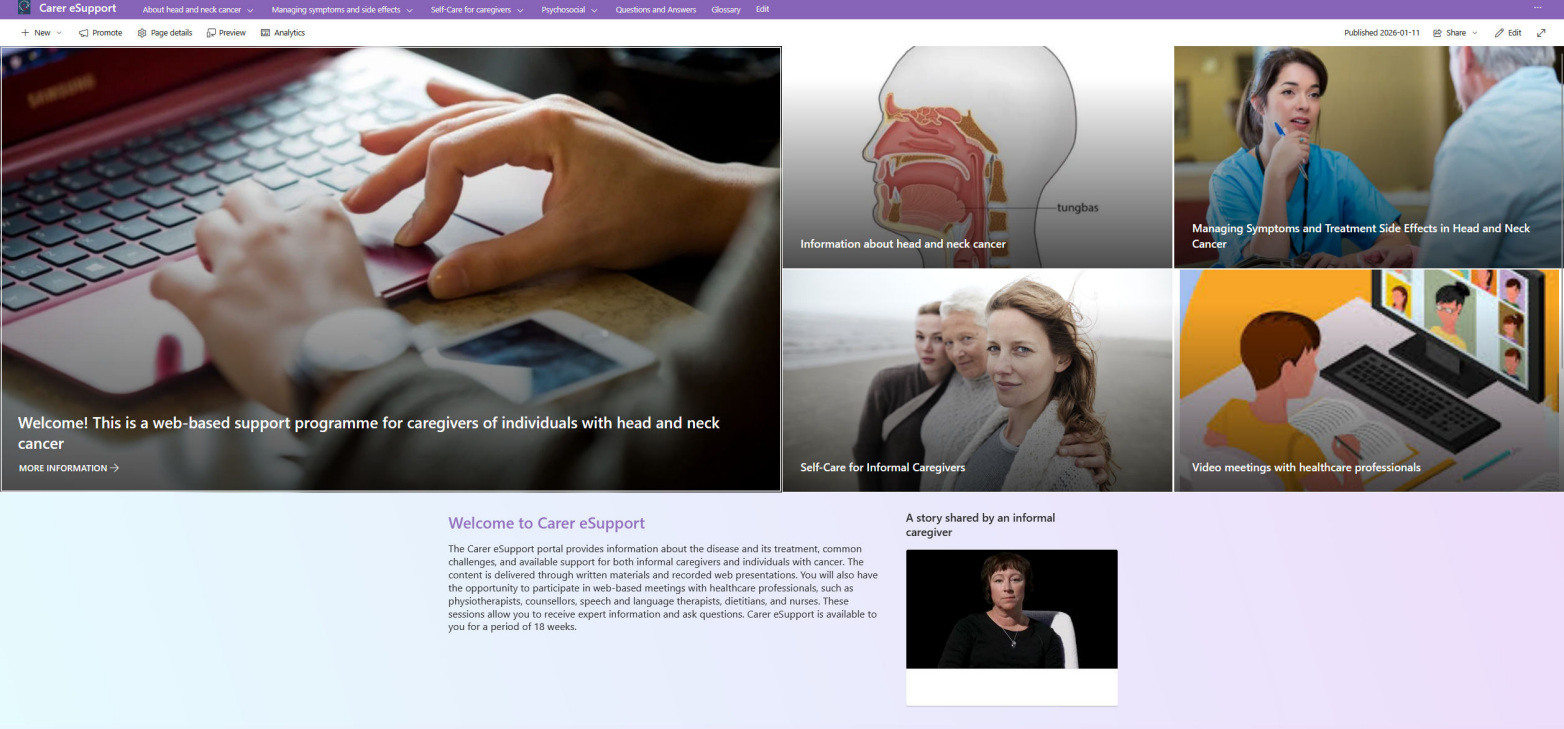
The main page of Carer eSupport.

The main page of Carer eSupport is organized into 5 modules: Information About HNC, Managing Symptoms and Side Effects of HNC, Self-Care for ICs, Psychosocial Support for ICs, and Interactive Video Meetings with HCPs and ICs (see Figure 1). In addition to these modules, the application features a Frequently Asked Questions section. This section addresses common queries related to HNC, caregiving, and the broader health care system. A glossary is provided to clarify medical terms and expressions, which helps ICs understand complex information with greater ease. Most of the content is provided in multiple formats, including lectures and written text, allowing ICs to choose the format that is easier for them. The application was initially developed in Swedish; however, the main page and module pages were explicitly translated into English for this paper to support a broader audience. To protect participants’ privacy, all screenshots were anonymized by blurring any faces that could lead to identification.

#### Information About HNC

**Figure 2.**
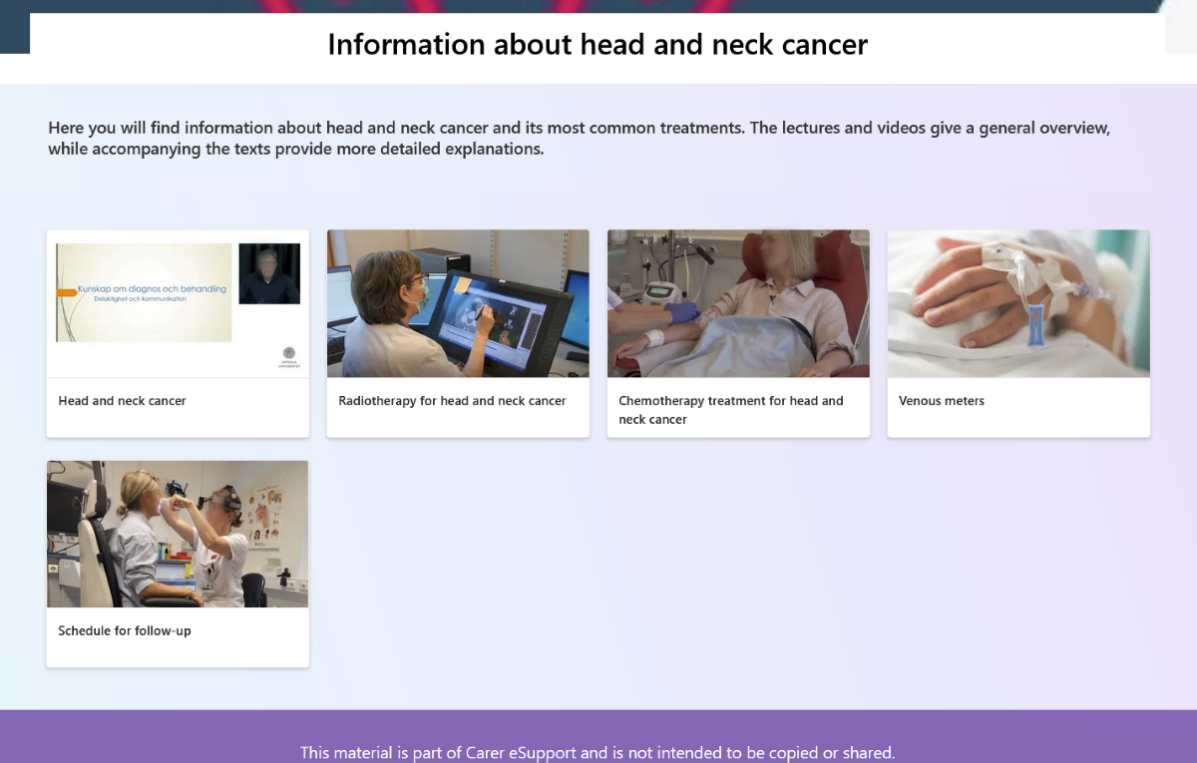
Module for information about head and neck cancer.

This module, as presented in Figure 2, provides a comprehensive resource on HNC and its common treatments to enhance the preparedness of ICs. It is designed to empower ICs by offering precise and detailed information on HNC and its treatment options, thereby improving their ability to manage the consequences of the disease and its treatment. The module addresses different aspects of HNC care and is anticipated to enhance a sense of competence and help ICs make autonomous and informed decisions aligned with a eudaimonic perspective.

The module is organized into 5 sections. The first section offers an overview of HNC, presenting essential information about the disease, its risk factors, and its typical progression. The following sections focus on treatment-specific details. One section provides a detailed explanation of radiation therapy, outlining its protocols, potential side effects, and strategies for managing these effects. Another section in this module is dedicated to chemotherapy, describing treatment regimens and common adverse reactions and offering practical advice for supporting patients during this modality. Moreover, the module includes guidance on using and managing venous catheters and presents the best practices to ensure patient safety. The final section, a follow-up schedule, explains the purpose and importance of each posttreatment visit and equips ICs with the knowledge necessary to support patients effectively during recovery.

#### Managing Symptoms of HNC and Side Effects of Treatments

**Figure 3.**
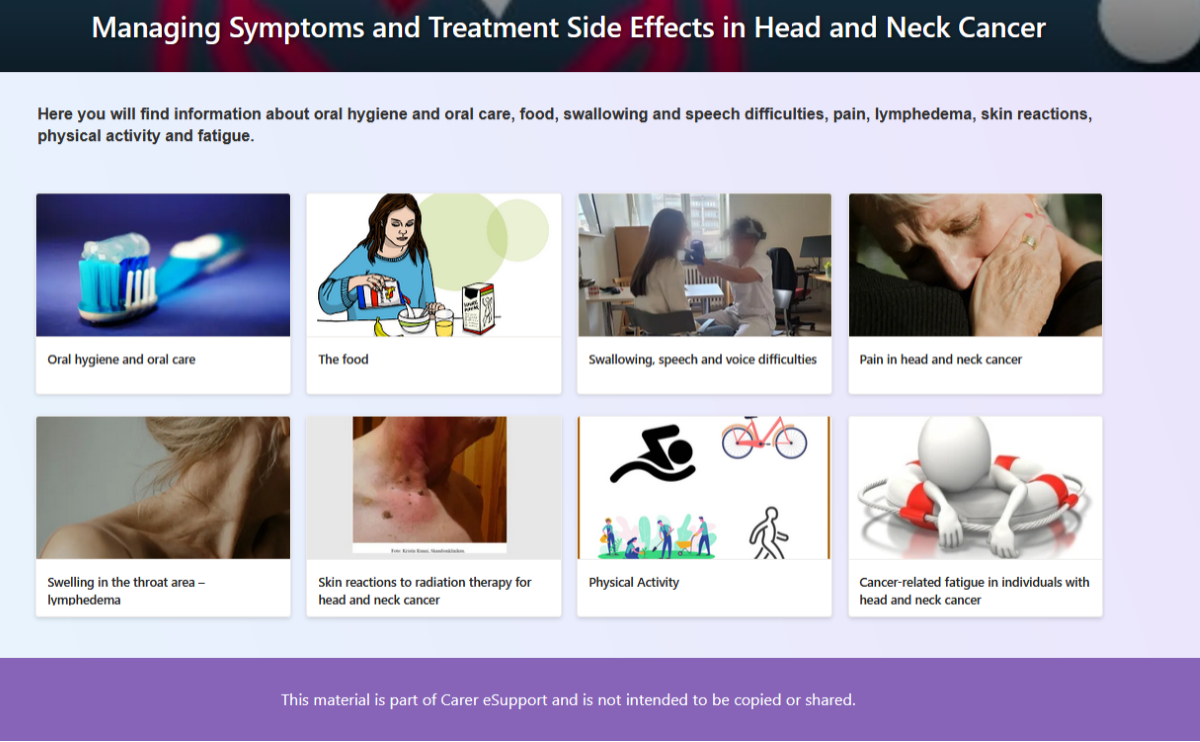
Module for managing symptoms and side effects.

This module is designed to better prepare ICs for the challenges of HNC by providing detailed guidance on managing the symptoms and side effects commonly experienced by patients (as presented in Figure 3). Structured into 8 sections, the module addresses key areas such as oral hygiene, providing detailed recommendations for maintaining oral health, especially after reconstructive surgery and radiotherapy. It includes guidance on nutrition, with a focus on dietary adjustments during treatment, along with resources like a recipe bank and information on feeding tubes.

Support is also offered for issues related to swallowing, speech, and voice, presenting practical strategies to manage these difficulties. The module includes targeted advice for pain relief and approaches to managing lymphedema, including recommendations for reducing swelling during or after radiation therapy. Strategies to mitigate skin reactions caused by radiation are outlined as well. Furthermore, it provides tailored approaches to maintain an active lifestyle that supports overall well-being. It offers insights and practical strategies to manage cancer-related fatigue, ensuring that ICs can support patients and themselves throughout treatment.

#### Self-Care for ICs

**Figure 4.**
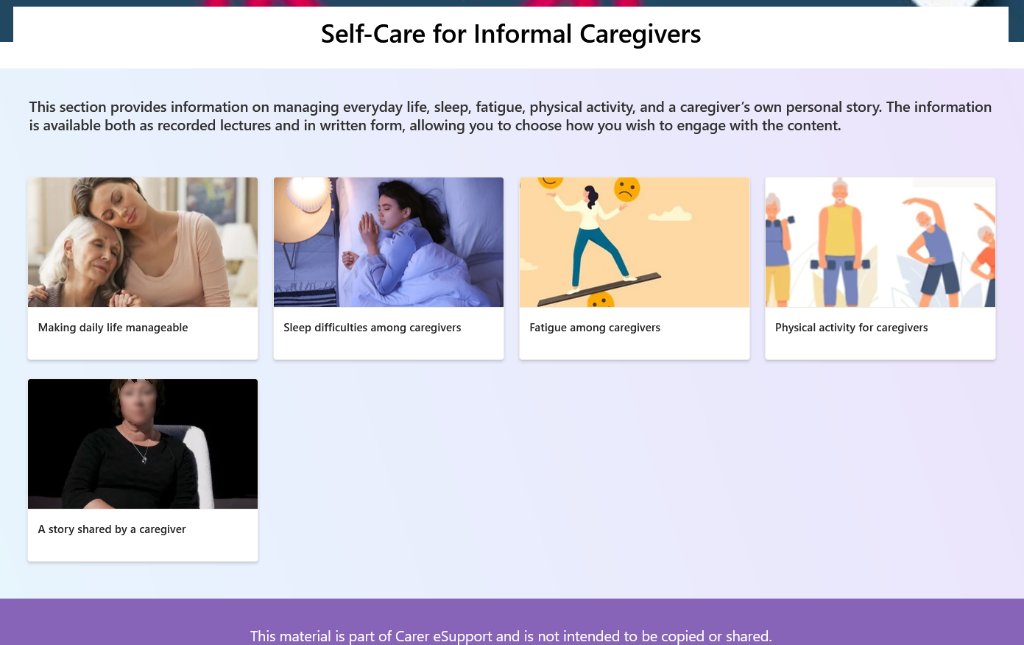
Module for informal caregivers’ self-care.

This module, as shown in Figure 4, supports the physical and psychological well-being of ICs by addressing everyday challenges such as managing routine tasks, sleep difficulties, fatigue, and the need for physical activity. The module is divided into 4 sections. It first provides strategies for adapting to life changes following an HNC diagnosis and then offers recommendations for overcoming common sleep issues. It also tackles exhaustion by introducing practical tools like a diary and a daily activity log to help balance responsibilities and self-care. It also emphasizes the benefits of maintaining an active lifestyle through regular physical activity.

Additionally, a personal story from an IC offers valuable insight into the emotional and physical challenges of caregiving, which may create empathy and a more profound understanding among ICs. The module promotes a eudaimonic perspective by strengthening ICs’ sense of competence and personal growth. Providing ICs with practical knowledge and strategies helps them feel more confident and capable, which may contribute to their well-being and resilience.

#### Psychosocial Support

**Figure 5.**
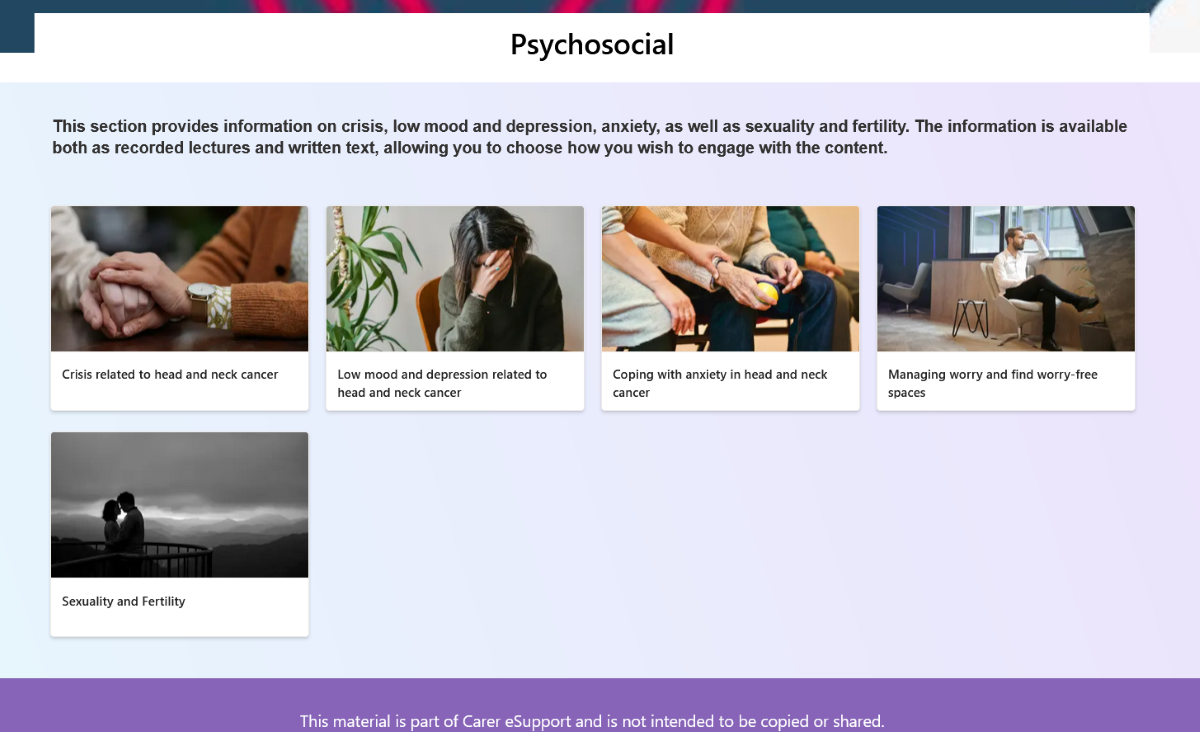
Module for psychosocial support.

The Psychosocial Support module, shown in Figure 5, is designed to enhance the emotional and psychological well-being of both ICs and patients by offering practical strategies for managing emotional health. This module is divided into 5 sections. It first addresses crisis reactions by providing coping strategies for the immediate psychological challenges during diagnosis and treatment. It then explores sadness and depression, offering guidance on recognizing and managing these emotions effectively. The following sections focus on anxiety, presenting practical strategies for managing worry and stress while also offering advice to create calm environments and concluding with an examination of the impact of cancer on sexuality and fertility, providing support for those affected by these issues.

From a eudaimonic perspective, each section is intentionally crafted to promote personal growth and to effectively prepare ICs to navigate the psychological challenges that accompany significant life changes. Beyond aiding emotional regulation, the module aims to empower ICs with a deeper sense of meaning and control in their roles. By reframing their experiences as opportunities for personal development, this approach may enhance resilience and improve their ability to cope with the demands of caregiving.

#### Video Meetings With HCPs

**Figure 6.**
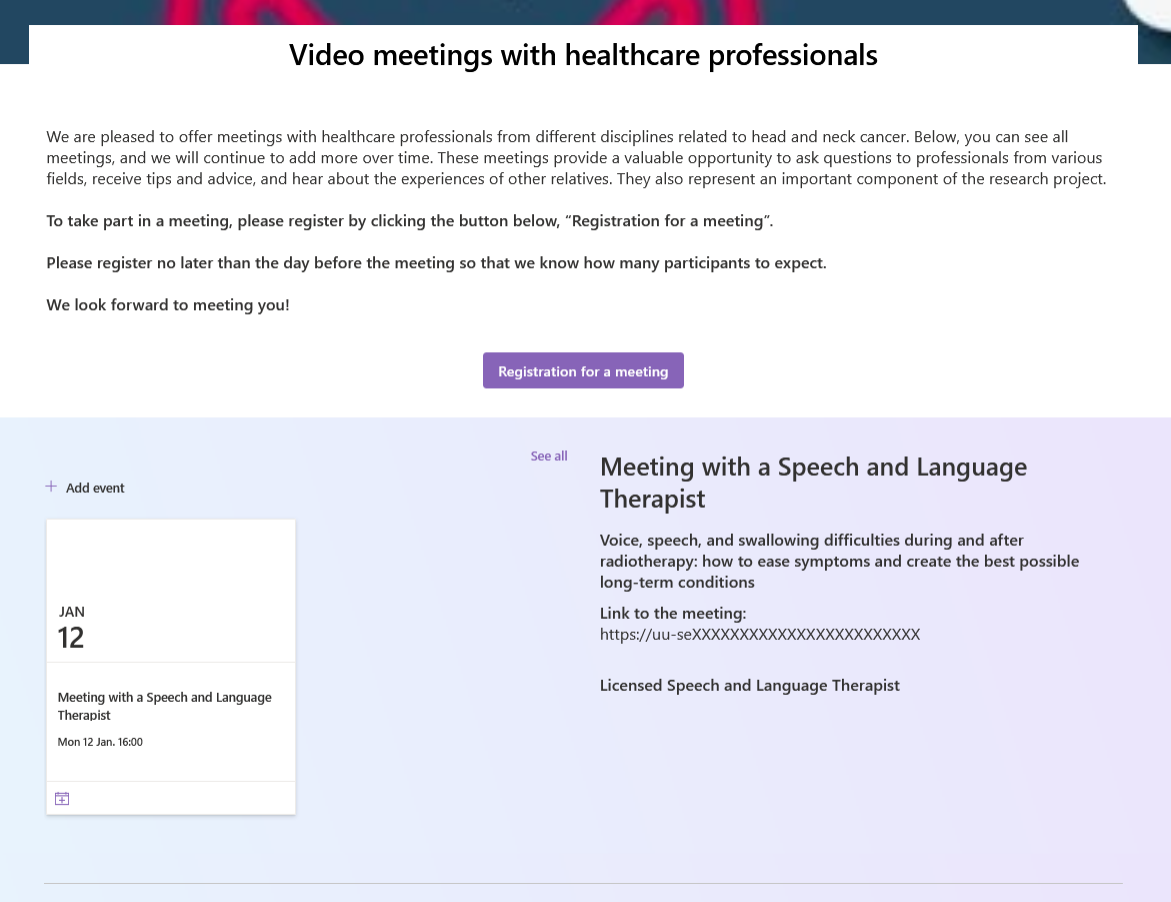
Module for video meetings with health care professionals.

This module allows participants to meet with HCPs from various fields related to HNC (Figure 6). These sessions allow ICs to ask questions, receive expert advice, and connect with others facing similar challenges. The module offers flexible participation options by allowing ICs to join meetings with or without video and remain anonymous if preferred, ensuring better accessibility and alignment with a eudaimonic perspective. This flexibility caters to diverse comfort levels with technology and fosters a safe space for open engagement.

Recognizing that meaningful community relationships are central to eudaimonic well-being, the design of these virtual sessions encourages ICs to share their experiences, ask questions, and engage in group discussions. Such interactions provide practical guidance while nurturing deep interpersonal connections that enhance emotional support and validation. Occasionally, sessions include short presentations or lectures, with details provided in advance on the application. ICs are encouraged to attend multiple meetings to reinforce learning and maintain access to relevant information. Additionally, they can email questions to HCPs anytime, receiving responses from nurses or other specialists, further strengthening their support system throughout caregiving.

### Result From the Pilot Study

**Figure 7.**
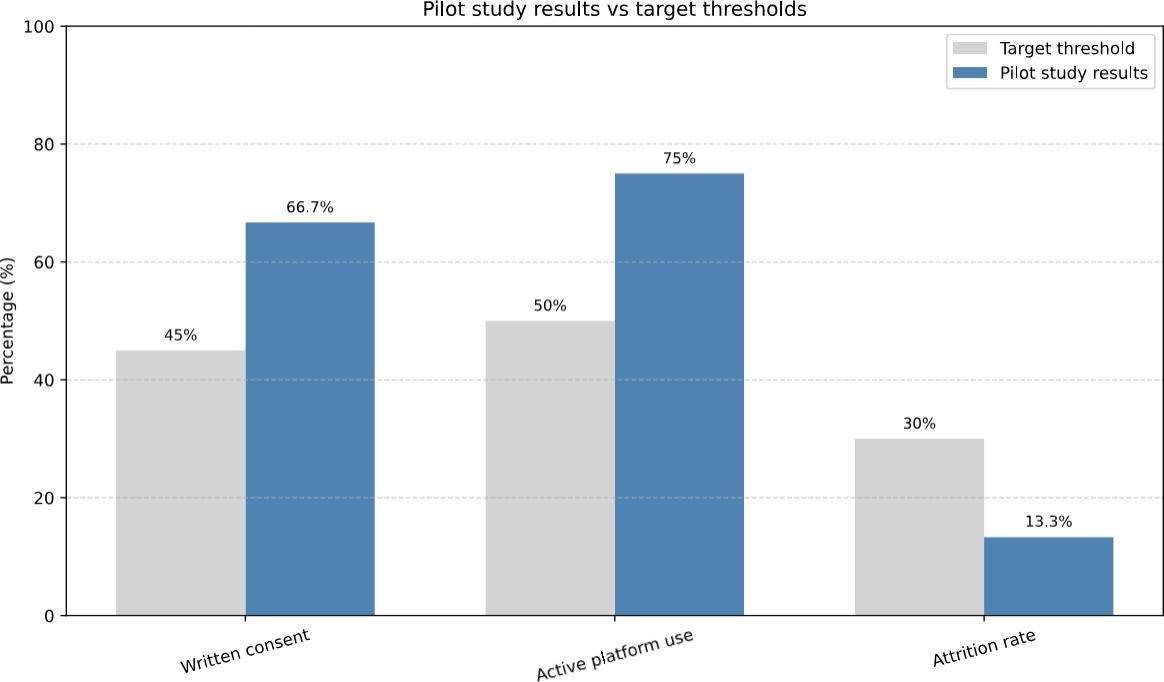
Pilot study results.

The results from the pilot study were satisfactory and met the established progression criteria with a good margin (Figure 7). Among the 30 participants, 20 (66.7%) ICs and 17 (56.7%) patients provided written consent. A total of 12 ICs were randomized to receive access to the application, and 9 out of these 12 (75%) successfully accessed the application. Additionally, 4 out of 30 (13.3%) participants discontinued their study participation.

### Challenges and Suggestions to Improve Usability and Accessibility of Carer eSupport

#### Overview

One week after sending the login details, we contacted participants who had not yet logged in to determine whether they required assistance. In this section, we summarize feedback from ICs regarding their experiences with Carer eSupport. Key challenges included time constraints due to the demanding nature of caregiving, communication barriers, login and authentication difficulties, limited IT knowledge, and inadequate access to proper IT infrastructure. To address these challenges and improve the application’s usability and accessibility, we implemented a range of solutions, as detailed below.

#### Demanding Nature of Caregiving

Due to their ongoing responsibilities, many ICs struggled to find the time and energy to learn new technology. Some of them noted that when their relatives were recently diagnosed with HNC and undergoing treatment, they had little opportunity to engage with the application. Follow-up contacts acted as gentle reminders, prompting them to log in once their situations stabilized. For example, one participant explained that although they could not access the application initially due to pressing concerns, a subsequent reminder enabled them to log in after their circumstances had calmed.

#### Communication Problems

Communication barriers with ICs also hindered their engagement with the application. Some ICs reported not receiving the initial email with their login details or finding it in their spam folder, which delayed access. To resolve this, we implemented a multichannel communication strategy by sending login details via email and SMS text messaging, making follow-up phone calls to clarify any issues, and offering real-time troubleshooting. It reduced delays in accessing the application and helped build trust with the users.

#### Login and Authentication Problems

Several ICs experienced difficulties logging into the application. Since Carer eSupport is built on Microsoft SharePoint, users with existing Microsoft accounts sometimes encounter conflicts, leading to errors when the wrong account is used for authentication. In addition, the 2-factor authentication process proved confusing for many users. To address these challenges, we updated the login instructions and tutorials and produced a video tutorial demonstrating the correct login procedure step by step. These improvements reduced errors to some extent for the users, and more users accessed the application without help from the support team.

#### Access to IT Infrastructure

Some participants faced challenges due to inadequate access to appropriate devices for using Carer eSupport. A few lacked a private computer and relied on work computers or smartphones, which sometimes imposed restrictions that prevented them from logging in. One participant, who relied solely on a smartphone, repeatedly had difficulty entering their username and password, resulting in failed login attempts and further complicating access. As we were unable to provide additional IT infrastructure during the study, we addressed these challenges through extensive telephone-based support. With this support, ICs were eventually able to access the portal. However, access would likely have been more straightforward if a computer or tablet had been available.

#### Limited IT Knowledge

The limited IT knowledge of ICs emerged as a barrier, particularly among older ICs or those with minimal technical experience. Navigating a new digital application can be overwhelming for ICs who are not tech-savvy. To mitigate this problem, we provided them with telephone support and developed clear and concise tutorials, demonstrating the login process and easy access to the application.

## Discussion

### Principal Findings

This study introduced an integrated HCD process that incorporated eudaimonic perspectives to develop the Carer eSupport application to improve ICs’ preparedness for caregiving and enhance their well-being. Our findings demonstrate that IT applications like Carer eSupport can be effectively developed through a multidisciplinary approach that integrates health care science and HCI methodologies. As a web-based application, Carer eSupport required complementary insights from HCI, mainly through an iterative HCD process, to ensure usability and user acceptance. This integration bridges conventional health care research with HCI, developing evidence-based and user-centered technologies, as supported by recent research [56]. To our knowledge, the Carer eSupport project is the first to use this strategy to support ICs of individuals with HNC.

A well-known challenge in HCI, and one central to this work, is balancing personalization with generalization [27,57]. Technologies that reflect ICs’ own situations are often perceived as more useful and engaging. For this reason, the HCD approach used to develop Carer eSupport focused on understanding ICs’ specific needs and caregiving contexts related to HNC. At the same time, practical constraints related to time, resources, and technical feasibility limit the degree of personalization that can be achieved [28,45]. HCI research recognizes that users may differ in their abilities, preferences, values, and life circumstances. HCD processes, therefore, begin with methods such as interviews and focus groups to identify human needs, which are then translated into requirements, personas, and scenarios. However, it is not feasible to design a separate system for each IC. Instead, design efforts focus on groups of ICs that share key characteristics while allowing variation within those groups. This tension was evident during the design of Carer eSupport. ICs preferred content reflecting their own HNC caregiving experiences, while HCPs emphasized the need for practical, sustainable solutions. The final design represents a pragmatic compromise, offering HNC-specific content at the user-group level while remaining relevant to a broad range of caregiving situations.

In this study, we presented the major modules and functions of the Carer eSupport application, explicitly incorporating the factors that may enhance the eudaimonic motivation of ICs. This was a challenging task due to the existing research’s broad and often vague definitions of eudaimonia. Previous research also highlighted that eudaimonic well-being is inherently subjective and can vary significantly from one individual to another [49,58]. For future research, it is essential to contextualize eudaimonic perspectives according to the user group and the contexts in which they interact with technology [17,43,49]. Otherwise, as noted in previous studies, eudaimonia risks becoming a buzzword without precise, practical implementation, despite its importance in technology adoption [27,41].

Our pilot study yielded promising results, with a 66.7% consent rate, a 75% successful login rate among randomized participants, and a low attrition rate of 13.3%. These outcomes met established progression criteria and confirmed the application’s readiness for a full-scale RCT. The study also provided important insights into usability challenges. ICs reported limited time due to demanding caregiving responsibilities, delays in receiving login details, difficulties with authentication, restricted access to suitable IT infrastructure, and limited digital literacy.

We provided multichannel communication via email, SMS text messaging, and follow-up phone calls to overcome these challenges. We also enhanced login instructions, created video tutorials, and provided additional telephone support. All these measures improved the application’s overall usability and accessibility, especially for first-time access to the application. The lessons learned from this study will be valuable in a broader context, contributing to the development and evaluation of complex interventions and to the refinement of RCT procedures for future web-based interventions like Carer eSupport.

As an important first step in the HCD process, understanding the user’s context and the use of technology is crucial for the smooth implementation of IT systems [33,59]. During the evaluation phase of an HCD process, real-world challenges often emerge that were not apparent at the start. For example, while assisting two ICs in logging into an application via smartphone, we discovered that they were attempting to juggle multiple tasks on a single device. The ICs were on a phone call and, at the same time, opened their email to look up their username and password. Afterward, they tried to access the application in another tab on the same smartphone. This multitasking led to repeated errors when entering their login credentials. The 2-factor authentication process, which requires ICs to retrieve and enter a code on the same smartphone, further complicates their access. When the support team asked whether they had access to an alternative device, such as a tablet or computer, the ICs said they had no other device available. After several attempts and extensive assistance from the support team, the ICs finally managed to log in to the application; however, it was not an easy process for them. Such cases highlight that while understanding user context is emphasized early in the HCD process, vital insights often emerge during evaluation. Such experiences, as highlighted in previous research, underscore the importance of iterative design improvements to ensure systems are more effectively aligned with users’ real-life circumstances [59,60].

Our integration of the HCD process and health care science, enriched by a eudaimonic perspective, offers initial guidelines for creating a framework that can be applied beyond the caregiving context. For technology designers and HCI researchers, especially those focusing on vulnerable groups, this study can inform the design and development of digital solutions that are both evidence-based and empathetic. However, it is worth noting that the study is limited to the pilot study. A complete evaluation of Carer eSupport can only be concluded once the full-scale RCT is completed. We look forward to exploring the full RCT outcomes, particularly regarding improvements in ICs’ preparedness for caregiving and their well-being.

### Conclusion

This paper presented the design and development of a web-based application that integrates eudaimonic perspectives within an iterative HCD process to prepare ICs for caregiving and improve their well-being. The pilot study results confirmed the application’s viability for an RCT, as evidenced by high consent, login, and retention rates. However, it highlighted significant user challenges, such as time constraints and technology-related barriers. Integrating the eudaimonic perspective into the application design also proved challenging due to the subjective and nuanced nature of the elements of eudaimonic motivation. Given the limited participant pool in this study, a more comprehensive evaluation is necessary to assess the relevance, effectiveness, and usability of Carer eSupport. This evaluation is currently underway in our ongoing RCT. Results from this study indicate that personalized, interactive web-based applications like Carer eSupport hold considerable potential to support ICs of individuals with HNC.

## Abbreviations

HCD: human-centered design
HCI: human-computer interaction
HCP: health care professional
HNC: head and neck cancer
IC: informal caregiver
RCT: randomized controlled trial
UTAUT: Unified Theory of Acceptance and Use of Technology
